# Consistency between stated and revealed preferences: a discrete choice experiment and a behavioural experiment on vaccination behaviour compared

**DOI:** 10.1186/s12874-015-0010-5

**Published:** 2015-03-12

**Authors:** Mattijs S Lambooij, Irene A Harmsen, Jorien Veldwijk, Hester de Melker, Liesbeth Mollema, Yolanda WM van Weert, G Ardine de Wit

**Affiliations:** National Institute of Health and the Environment, Centre for Prevention and Health Services Research, P.O. Box 1 3720, Bilthoven, BA The Netherlands; National Institute of Health and the Environment, Centre for Infectious Disease Control, P.O. Box 1 3720, Bilthoven, BA The Netherlands; Julius Centre for Health Sciences and Primary Care University Medical Center Utrecht, P.O. Box 85500, Utrecht, GA 3508 The Netherlands; Maastricht University, Work & Social Psychology, P.O. Box 616, Maastricht, MD 6200 The Netherlands

**Keywords:** DCE, Predictive value, Stated preferences, Revealed preferences, Vaccination, Hepatitis B

## Abstract

**Background:**

Discrete Choice Experiments (DCEs) are increasingly used in studies in healthcare research but there is still little empirical evidence for the predictive value of these hypothetical situations in similar real life circumstances. The aim of this paper is to compare the stated preferences in a DCE and the accompanying questionnaire with the revealed preferences of young parents who have to decide whether to vaccinate their new born child against hepatitis B.

**Methods:**

A DCE asking parents to decide in which scenario they would be more inclined to vaccinate their child against hepatitis B. The stated preference was estimated by comparing the per respondent utility of the most realistic scenario in which parents could choose to vaccinate their child against hepatitis B, with the utility of the opt-out, based on the mixed logit model from the DCE. This stated preference was compared with the actual behaviour of the parents concerning the vaccination of their new born child.

**Results:**

In 80% of the respondents the stated and revealed preferences corresponded. The positive predictive value is 85% but the negative predictive value is 26%.

**Conclusions:**

The predictive value of the DCE in this study is satisfactory for predicting the positive choice but not for predicting the negative choice. However, the behaviour in this study is exceptional in the sense that most people chose to vaccinate. Future studies should focus on behaviours with a larger variance in the population.

**Electronic supplementary material:**

The online version of this article (doi:10.1186/s12874-015-0010-5) contains supplementary material, which is available to authorized users.

## Background

Discrete Choice Experiments (DCEs) are increasingly used to better understand how patients make health care decisions [1], (e.g. [2,3]). A critique of DCEs is that their predictive value may be restricted as they measure stated (i.e. costless and effortless) preferences and actual behaviour may differ from these stated preferences [1,4-6].

A basic assumption of DCEs is that people base their choices on latent preferences and seek to maximize their utility [7,8]. In most DCEs the respondents are asked to imagine that they find themselves facing a choice and to reveal which alternative they prefer. It is assumed that the stated choices are congruent with decisions that would be taken in similar situations in real life.

Comparisons between stated and revealed preferences in DCEs have mainly considered willingness to pay (WTP), and often demonstrate a discrepancy between the stated and revealed preferences [9-13]. However, WTP is just one type of potential behaviour that is studied in DCEs. Comparing stated preferences with actual behaviour other than paying for a good or service may further explore the predictive power of the DCE-method.

In this study, parents were asked to decide whether to vaccinate their new born child against hepatitis B. At the time of the study, the hepatitis B vaccine had not yet been introduced universally within the Dutch National Immunization Program (NIP). We hypothesized that stated preferences would correspond with actual choice behaviour (revealed preferences). Traditionally, all vaccinations have had very high uptake in the Netherlands (>95-97% of population is covered). However, recent vaccination campaigns showed uptake rates of around 50% (for HPV vaccination) and are increasingly scrutinized in public discussions (i.e., influenza vaccination).

The current study was conducted amidst growing concerns about the acceptance of vaccination against human papillomavirus (HPV) and H1N1 Influenza A in the Dutch national immunization program (NIP) [14]. These examples showed that people no longer accepted the advice from the regulatory bodies without questions. As a consequence, it is no longer straightforward that people accept all vaccines that are offered in the context of a NIP. After the decision to introduce universal hepatitis B-vaccination in the NIP was taken, the Ministry of Health commissioned this study. At the time of the study, only risk groups (new borns with a parent from an endemic country, drugs users, men having sex with men and commercial sex workers) were offered hepatitis B-vaccination, while currently all new borns were eligible for hepatitis B vaccination. The hepatitis B-vaccine is part of a combination vaccine consisting of diphtheria, pertussis, tetanus, polio and *Haemophilus influenzae* type b and hepatitis B vaccine (DTaP-IPV-Hib-HepB or 6-valent vaccine). In order to test which factors may influence the decision whether or not to accept this 6-valent vaccine, we conducted a DCE. After completing the DCE, we offered the same parents the 6-valent vaccine for their child, prior to the universal introduction of this vaccine.

The aim of this paper is to study the congruence between parents’ stated and revealed preference for vaccination of their new born child against hepatitis B.

## Methods

### Design of the choices

Table 1 presents all attributes, levels and the corresponding questions from the questionnaire including the recoding for further analyses. The selection of the attributes was based on focus group interviews from a previous study with parents of new born babies concerning the hepatitis B vaccine [15], literature review, and expert interviews.

**Table 1:** **Attributes and levels of the DCE, along with corresponding questions from the questionnaire**

**Attributes**	**Variable name**	**Levels (coding in analyses)**
Infection Risk without vaccination	INFR	(-1) unknown to you
		(1) 1 to 500
Side effects of vaccination	SIDE	(-1) unknown to you
		(1) comparable to regular vaccination
Possibility to choose for hepatitis B or not	CHOICE	(-1) you cannot choose whether to vaccinate your child with or without the hepatitis B vaccine
		(1) you can choose whether to vaccinate your child with or without the hepatitis B vaccine
Source of information that the vaccine is safe	NIH	(0) GP
	WFC	(-1) a folder by the National institute of health
		(1) the child welfare centre
Source of information that the vaccine causes problems (a child has been hospitalized after getting the vaccine)	SCM	(0) the news on TV
	ACQ	(-1) social media (e.g. facebook, twitter, blog)
		(1) acquaintance
Attitude of social environment (Number of friends getting their child vaccinated)	FRI	(-1) none of your friends
		(1) all of your friends

The first attribute (Infection Risk without vaccination, in Table 1) refers to the importance of knowledge on the a priori chance of getting hepatitis B infection without vaccination. This was included in the design because Brown et al. [5] found that the protection level of the vaccine influenced the preference of mothers of daughters who were eligible for HPV vaccine [5]. The second attribute (Side effects of vaccination) addressed the importance of knowledge on the risk of side effects following vaccination. This attribute was included because in earlier studies it was found that the risk of side effects affected girls’ preferences for vaccination [4,16]. The third attribute (Possibility to choose for hepatitis B or not) was included because parents indicated in the focus group interviews that this was important [15]. We included an attribute on the freedom of choice for parents between a vaccine with hepatitis B (DTaP-IPV-Hib-HepB) or without hepatitis B (DTaP-IPV-Hib). This implies that an active choice can be made for a vaccine with or without the hepatitis B component (see also [17]).

Paulussen et al. [18] emphasized that the reliability and trustworthiness of the health care practitioners might have an important influence on the decisions of parents whether to have their child vaccinated or not, this was confirmed by our experts. Therefore, an attribute (Source of information that the vaccine is safe) that consisted of three different sources stating that the vaccine was safe, was included in the DCE. Previous research found indications that various forms of information exchange affect the uptake of vaccination.

Subsequently, we included an attribute on various informal sources spreading a rumour that a child was taken to hospital after being vaccinated (Source of information that the vaccine causes problems (a child has been hospitalized after getting the vaccine)). This attribute was selected because it was found that after the introduction of vaccination against cervical cancer for 12-year-old girls (with a catch-up campaign for girls aged 13–16 years) in the Dutch NIP in 2009, national media attention negatively affected the uptake of vaccination [19]. Van Keulen et al. described how information exchange by girls on the internet led to fear of vaccination [20] and this was confirmed by our experts. Finally, peer groups are known to affect attitudes towards vaccination [21]. Therefore, an attribute (Attitude of social environment) on the vaccination behaviour of friends and relatives was introduced.

### Design of DCE, introduction of the scenarios

In total, the full factorial set consisted of 2^4^*3^2^ = 144 scenarios. We used random foldover to pair scenarios into choice sets. This means that each scenario was randomly paired with another scenario from the full factorial set, resulting in 72 choice sets. The 72 choice sets were blocked, resulting in 18 unique sets of four choice sets. We repeated this foldover process to create 2000 unique questionnaires.

The DCE consisted of four choice sets per respondent; each choice set contained two unlabelled scenarios. Parents were asked to choose between the two scenarios. Subsequently, they were asked to state how certain they were about their choice on a 10-point scale anchored by the statements “I am certain that I would not have my child vaccinated in this situation” and “I am certain that I will have my child vaccinated in the chosen situation”. The choices were introduced with a detailed description of the case. An illustration of the DCE is displayed in Additional file 1.

The DCE was part of a questionnaire that included questions on psychological and social characteristics. The questionnaire included questions on the respondents’ estimation of the protection of the vaccine, side effects, opinion on combining standard vaccination with hepatitis B vaccination, their use of both formal and informal sources of information, and on vaccination behaviour of others in their social network. The questionnaire also gathered information on socio-demographic characteristics of the respondents.

### Study population

The study population consisted of parents with a new-born younger than two weeks that was not yet eligible for hepatitis B vaccination (i.e. new-borns that did not belong to risk groups). A random sample of 2000 addresses of these new-borns was taken from a national database that is used to send invitations to parents for the NIP (Praeventis database). Vaccination status is registered in this database as well. The parents did not receive specific additional information on hepatitis B, which is in line with current standards within the NIP. In the questionnaire, respondents were asked whether they wanted to participate in a follow up study, without reference to the character of this follow-up study. People who indicated their willingness to participate in the follow-up study received a letter after four weeks with the offer to have their child vaccinated against hepatitis B. They were free to reject the offer. The letter was accompanied by additional information, an informed consent form and a letter to their GP (please see also [22] for elaborate description of the process of data collection). Parents who opted for the 5-valent hepatitis B vaccine, enrolled in the regular program and did not need a special voucher.

### Observation of revealed preferences

For the parents who returned the informed consent form, we extracted vaccination decision (with or without hepatitis B) from the National Praeventis database of the NIP. As there is some fluctuation in the timing of uptake of the first vaccination within the NIP, the actual vaccination behaviour was checked 2.5 months after the invitations were sent, to make sure that most babies would have had their first vaccination. The Institutional Review Board of the University Medical Centre Utrecht approved the study.

### Analyses

#### Step 1: computing individual preference to accept vaccination or not, based on DCE

The answer to the question how certain the respondent was that their child would be vaccinated after making the choice between scenarios was used to identify the opt out. When the score on the 10-point scale was below 6, the respondent was coded to have chosen the opt-out in that choice set.

The subsequent data from the DCE were analysed with mixed logit, using NLogit 5.0. The attribute levels were effects coded (see Table 1 for precise coding). The test for the necessity of random parameters, based on log likelihood improvement, resulted in the following parameters being set random, assuming a normal distribution: The effectiveness of the vaccine, the side effects of the vaccine and the source of information that the vaccine causes problems (both social media and acquaintance). The following equations were tested:

$$ \begin{array}{l}{V}_{A/B}={b_0}_{,i}+{b_1}_{,i} INFR+{b}_{2,i} SIDE+{b}_3 CHOICE+{b}_4NIH+{b}_5WFC+{b}_{6,i}SCM+{b}_{7,i}ACQ+{b}_8FRI\\ {}{V}_{opt- out}=0\end{array} $$

V is the deterministic utility of the choice and can be calculated as the observed utility which is the sum of β0 (the intercept) and β1 – β8, which are the attribute estimates that indicate the relative importance of each attribute.

The model presented in the equation was estimated. The potential vaccination coverage rate was estimated at population level for the following vaccine scenario: the effect of the vaccination is known (INFR = 1), the risk of side effects is comparable to regular vaccination (SIDE = 1), people have no choice (CHOICE = −1), the National Institute of Health advices to use the vaccine (NIH = 1), the child welfare centre advices to use the vaccine (WFC = 1), a social media notification of hospitalization of a child after vaccination (SCM = 1), this story is also told by acquaintances (ACQ = 1) and all friends take the vaccine (FRI = 1).

The potential coverage rate can be calculated as 1/(1 + exp^-v^). Since V includes random parameters, the standard deviation of those parameters should be accounted for [8,23]. The value of the random parameters was determined by taking 10,000 draws from a normal distribution with a mean and standard deviation (SD) for that particular random parameter (i.e., the mean and SD values were retrieved from the mixed logit model). For every draw of the random parameter value, the observed utility ‘V’ as well as the potential coverage rate was calculated. The average of the 10,000 calculated potential vaccination coverage rates was reported.

Second, the individual-level beta values were stored by Nlogit software for all random variables (i.e., the intercept, effectiveness, side-effects, and the source of information). These individual beta values were then used to compute the utility for the vaccine scenario described above. This resulted in a utility score for this particular scenario for every respondent separately. Subsequently, the individual utility scores were compared to the utility of the opt-out option. If the utility of the scenario was larger than the utility of the opt-out, the respondent was assumed to opt for vaccination, and vice versa.

#### Step 2: comparing the predictions with actual behaviour

The individual-level stated preferences with respect to hepatitis B vaccination from step 1 were compared to the observed behaviour in a 2×2 table. Predictions were marked as correct, when the predicted acceptance of hepatitis B vaccination by the parents corresponded with the children’s vaccination status (yes/no) as recorded in the database of the NIP.

### Positive predictive value and Negative predictive value

In order to interpret the value of the predictions, we calculated the positive predictive value (PPV) and negative predictive value (NPV). The terms “positive predictive value” and “negative predictive value” are mostly used in diagnostic testing [24]. The PPV of a test is defined as the proportion of people with a positive test result who actually have the disease. The NPV of a test is defined as the proportion of people with a negative test result who actually do not have the disease. In this study, the PPV expresses the proportion of respondents who were congruent in their stated and revealed preference in accepting the vaccination; the NPV expresses the proportion of respondents who were congruent in their stated and revealed preference in rejecting the vaccination.

## Results

### Study population and response

Of the 2000 questionnaires, 906 were returned (response rate = 45.3%). Six respondents were not included in the analyses because they stopped completing the questionnaire already at the demographic variables. An additional four were not included because they had not answered any of the choice sets. Eleven respondents with missing values on some but not all of the choice sets were included. This resulted in 7,132 observations (scenarios assessed) in 896 respondents.

The mean age of the respondents was 31.5 years, (range 16–48 years) (see Table 2). Of the respondents 82% were female. Of all respondents, 90% reported no conviction or religion that influenced their opinion on vaccination, while 6% reported that their religious conviction affected their opinion on vaccination. In the Netherlands, about 1.3% of the population belongs to orthodox protestant subgroups [25]. These groups refuse vaccination because it presumably interferes with God’s will [26,27]. Finally, 4% reported that other convictions influenced their attitude to vaccination (2% homeopathy, 1% nature medicine or anthroposophy, 1% “other conviction”). These groups refuse vaccination because of the potential harm the vaccination presumably causes to the body. Almost half (48%) of the respondents reported positive previous experiences with vaccination, while about 1% of the respondents reported negative experiences with vaccination.

**Table 2:** **Descriptive statistics sample**

	**Mean (s.d.)**	**%**
Gender female		81.8
Age (years)	31.5 (4.8)	
Education primary		10.2
Education secondary		39.3
Education higher		50.4
No religious conviction		90.0
Religious conviction that affects vaccination choice		6.0
Other conviction that affects vaccination choice (homeopathy, nature medicine or anthroposophy)		4.0
Newly born child firstborn child = yes		52.2
Bad experience vaccination first child (for those eligible)		1.2

### Results step 1: the model on DCE data

In total 3,566 choice sets (7,132 scenarios) were used for the analysis. Table 3 presents the results of the mixed logit model of the DCE. The middle column presents the beta values of the mixed logit analyses. The right column of Table 3 presents the standard deviations (assuming a normal distribution) for the parameters with random slopes (intercept, the infection risk without vaccination, side effects and the source of the story of hospitalization of a child). The fact that these standard deviations are significant implies that preference heterogeneity is present for these attributes amongst the respondents.

**Table 3:** **Results mixed logit, predicting preference of vaccination behaviour in DCE**

	**b (s.e.)**	**Sd for random parameters (s.e.)**
Constant	-2.77 (0.71)**	4.55 (1.06)**
Infection Risk without vaccination		
Unknown to you	-0.34	
1 to 500	0.34 (0.05)**	0.32 (0.29)
Side effects		
Unknown to you	-0.39	
Comparable to regular vaccination	0.39 (0.06)**	0.67 (0.18)**
Choice		
No choice offered for hepatitis B or not	-0.18	
Choice offered for hepatitis B or not	0.18 (0.04)**	
Source of information that the vaccine is safe:		
General Practitioner	-0.06	
National Institute Health	-0.19 (0.06)**	
Child welfare centre	0.25 (0.06)**	
Source of story hospitalization of child:		
TV news	0.38	
Social media	-0.20 (0.05)**	0.12 (0.29)
Acquaintance	-0.18 (0.06)**	0.64 (0.25)*
Attitude of social environment:		
No friend vaccinates	-0.70	
All friends vaccinate	0.70 (0.06)**	
Log likelihood	-3464.02	
AIC	1.97	

** = p<0.01; *p<0.05 (log likelihood null model -3897.87).

All the coefficients of the betas are significant (p < 0.01). The largest coefficient was found for attitude of social environment (b = 0.70; s.e. = 0.06), so friends vaccinating their children strongly affect the choice for vaccination among respondents. Two other important factors that affect the decision to vaccinate are ‘knowledge that the risk of possible side effects is comparable to regular vaccination’ (b = 0.39, s.e. = 0.06) and ‘knowledge of the a priori risk of getting hepatitis B without vaccination’ (b = 0.34, s.e. = 0.05). Furthermore, informal sources of information reporting on problems with the vaccine affect vaccination decisions. When social media report a child being admitted into a hospital after vaccination or when an acquaintance reports this (b = −0.20, s.e. = 0.05 and b = −0.18, s.e. = 0.06, respectively), the willingness to vaccinate is less than when the story originates from official news sites.

Next, people prefer to have a possibility to choose between a vaccine with or without the hepatitis B component (b = 0.18, s.e. = 0.04), but this is less important in the vaccination decision than the other characteristics. Concerning the (formal) sources of information that the vaccination is safe, all sources have a relatively small impact on the choice of the respondents. However, of the formal sources, the clinician of the child welfare center has some positive influence compared to the GP (b = 0.25, s.e. = 0.06), while communication by the National Institute of Public Health has a negative influence on the willingness to have a child vaccinated, compared to communication by the GP (b = −0.19, s.e. = 0.06).

### Results step 2: comparing the stated and revealed preferences

The real uptake (revealed preferences) of the Hepatitis B vaccine in the sample is 84% (Table 4). On population level, a coverage rate of 76% (95% CI: 44%-94%) was estimated for a vaccination scenario that closely resembles the situation in real life.

**Table 4:** **Comparison of stated and revealed preferences**

		**Stated preference**		
		***Not vaccinate Hep B***	***Vaccinate Hep B***	
***Revealed preference***	***Not vaccinate Hep B***	6	33	39 (16%)
	***Vaccinate Hep B***	17	191	208 (84%)
		23 (9%)	224 (91%)	247

(0 = choice against vaccination; 1 = choice for vaccination).

Cohen’s kappa = 0.09 (approx. T = 1.54, ns).

Table 4 presents the cross table of the computed stated preference and the revealed preference of the 247 respondents we included in the analyses. The table shows that 224 responders (91%) have a higher utility for the vaccination scenario than for the opt-out, and thus a stated preference of choosing for vaccination. For 23 responders (9%), the utility for opt-out was larger than the utility for the scenario.

The main diagonal of Table 4 represents the number of respondents with correspondence between stated and revealed preferences. Overall, 197 (191 + 6) decisions were predicted correctly (80%). In 33 cases (13%), vaccination was predicted but not received. In 17 cases (6%) it was predicted that parents would not have their child vaccinated against hepatitis B, while in reality they accepted the vaccination offer.

Table 4 further shows that a majority of people accepted the Hepatitis B vaccination (84%), while a minority deviated from this behaviour and did not get the vaccination (16%). This asymmetry in the distribution of the vaccination behaviour is the cause of the Kappa measure being non-significant, despite the percentage of correct predictions [28,29].

In our case, the PPV is 85% (191/224 = 85%) and the NPV is 26% (6/23 = 26%).

## Discussion

The aim of this paper was to study the predictive value of the DCE-technique by comparing stated with revealed preferences among parents deciding whether or not to vaccinate their new born child against hepatitis B. For 247 parents, predicted vaccination behaviour was compared with their actual choices regarding vaccination of their new born against hepatitis B. In 80% of the respondents, their actual behaviour was predicted correctly by the stated preference DCE. The positive predictive value was 85% and the negative predictive value was 26%.

Previous research in health care that compares stated and revealed preferences of patients, was conducted by Ryan and Watson [30,31]. That study compared the results of a DCE that used multiple hypothetical scenarios to the real life choice to participate in chlamydia infection screening. The study found 81% correspondence between the stated and revealed preferences. The authors concluded that their predictions overestimated real behaviour and that more research was needed [31]. The percentage of correct predictions in the current study is similar to that study, and in direct comparison between the stated and revealed preferences, we also find an overestimation (predicted 91% vs 84% observed), but in the aggregate estimated uptake, we found an underestimation (76% estimated uptake).

In current DCE research, the different attributes are in general interpreted as attributes of goods or of a product that respondents trade off against one another. We included not only attributes of vaccination itself (such as side effects and effectiveness), but also attributes that refer to the physical (location of vaccination) and social environment in which the vaccination is given. Although the latter attributes may be relevant in the decision of parents, they cannot be influenced by health policy makers that aim to offer a vaccination programme with high acceptance levels. Therefore, our attributes should be interpreted as factors that influence decisions that respondents make, rather than as characteristics that can be traded among each other.

We obtained data and informed consent to compare the stated and revealed preferences for only 247 of 906 (27%) respondents. A selection bias in this group may have distorted our results. We compared the group of respondents that consented to participate with the group of respondents that did not agree to participate in the second stage of the study with regard to educational level, presence and number of other children in the family, and presence of (religious) convictions (not shown). Both groups did not differ with regard to the presence of (religious) convictions or number of previous children, but the group that allowed us to study revealed preferences was more highly educated. Additional analyses (not shown) with a mixed logit model of the sample of people who allowed us to compare their stated preference with their revealed preference, show that the general preference of this group is more positive towards vaccination.

In this study we used a dual-response response design with a scale to indicate the respondents’ certainty of the answer. Research found no differences between the dual-response choice design and a single-step opt-out design [32]. Because of the scaled certainty question, we had to make a choice for the dichotomization of the scores into either opt-in (acceptance of vaccination) and opt-out (refusal of vaccination). We decided to make the cut off at the centre of the 10 point-scale (between 5 and 6). In order to test how this affected the answers, we analysed the answers leaving out the choices with a value 5 or 6 on the scale (analyses not shown). We found that the general answer patterns remained similar.

Even though the percentage of correct predictions is encouraging, the respondents turned out to behave more similarly than expected. The large majority chose to have their child vaccinated against hepatitis B. In 7% we predicted that people would not get the vaccination with Hepatitis B, while they did, whereas in 13% we predicted that they would get the vaccination, while they did not (Table 4). A possible explanation for people unexpectedly accepting the vaccination could be that participation in the study encouraged the respondents to inform themselves about the topic and change their point of view.

A possible explanation for people unexpectedly not accepting the vaccination may lie in the disturbance caused by the amount of study-induced information that was included with the request to participate in the second phase of the study. At the instigation of the Institutional Review Board, the researchers had to add a letter to the second phase of the study (where the real vaccine was offered), asking parents to inform their family doctor when they chose to vaccinate their child. Also, despite the fact that the hepatitis B vaccine has been safely administered to millions of new-borns worldwide, the Institutional Review Board only allowed this study when information material accompanying the offer to vaccinate emphasized that insurance covered possible harm caused by the study. This may have discouraged some parents who initially may have intended to accept the vaccination offer.

Although this study adds to the knowledge on the relationship between stated and revealed preferences, it does not provide a definite answer with regard to external validity of DCEs. The behaviour in this study was very asymmetric (most people chose the vaccination) only yielding evidence of acceptable predictive validity in a situation of a population largely accepting the vaccination. If possible, our study should be repeated with a health behaviour that shows more variation in a population. This could for instance be tested in HPV vaccination, were acceptance levels are around 50% in the target group of adolescent girls [33].

## Conclusions

The added value of this study is the unique combination of data on the stated preferences of respondents and observations of their actual behaviour. We found some evidence that the stated preferences we measured were related to the actual behaviour of respondents, and that the positive predictive value was satisfactory. However, due to the skewed distribution in the behaviour, the negative predictive value was low. To further study the relationship between stated and revealed preferences, a similar study on a different type of (health) behaviour should be conducted, preferably on behaviour that shows more variance within the population.

## Additional file

Additional file 1:
**Example of introduction and choice set.**

## Abbreviations

DCE: Discrete Choice Experiment
HPV: Human papillomavirus
NIP: Dutch National Immunization Program
PPV: Positive predictive value
NPV: Negative predictive value
