# Supplementary material for: Consistency between stated and revealed preferences: a discrete choice experiment and a behavioural experiment on vaccination behaviour compared
Source: BMC Med Res Methodol. 2015 Mar 12;15:19. doi: 10.1186/s12874-015-0010-5 (PMC4359569; doi:10.1186/s12874-015-0010-5)
Supplement: Additional file 1: — Example of introduction and choice set. [file 12874_2015_10_MOESM1_ESM.doc]

**Additional file 1 Example of introduction and choice set**

*We will present several situations to you concerning Hepatitis B vaccination. The information in the grey column is similar across all situations. The two other columns contain small differences. We ask you to choose whether you prefer to have your child vaccinated in situation 1 or in situation 2. Subsequently, you can indicate how certain it is that you actually would have your child vaccinated in the chosen situation. It is important to know that not all situations occur in real life.*

*Please try to imagine that your child is about two months old and you are at the child welfare center for the first vaccinations. Instead of the regular vaccination, you are offered a vaccination including a vaccine against Hepatitis B. You need to decide to have your child vaccinated in situation 1 or 2. Which situation would you choose?*

**Choice** 1

|  | Situation 1 | Situation 2 |
| --- | --- | --- |
| The chance without vaccination that your child will get Hepatitis B is… | 1 to 500 | unknown to you |
| The chance of additional side effects because of the hepatitis B vaccination is… | unknown to you | comparable to regular vaccination |
| There is a vaccination with hepatitis B and a vaccination without hepatitis B … | you are not allowed to choose whether your child gets the vaccine with or without the Hepatitis-B vaccine | you can choose whether your child gets the vaccine with or without the Hepatitis-B vaccine |
| You read or hear that the vaccine is safe through… | your GP | the child welfare centre |
| You read or hear that a child is hospitalized after receiving a hepatitis-B vaccination through… | the social media (e.g. facebook, twitter, blog) | the TV news |
| To have his or her child vaccinated against hepatitis B … | … is chosen by none of your friends | … is chosen by all your friends |
| When would you prefer to have your child vaccinated? |  scenario 1 |  scenario 2 |

2. How large is the chance that you really would have your child vaccinated in the scenario that you chose?

| I know for sure that I would not have my child vaccinated in the chosen scenario | 1 | 2 | 3 | 4 | 5 | 6 | 7 | 8 | 9 | 10 | I know for sure that I would have my child vaccinated in the chosen scenario |
| --- | --- | --- | --- | --- | --- | --- | --- | --- | --- | --- | --- |
